# Advances in Molecular Pathology of Obstructive Sleep Apnea

**DOI:** 10.3390/molecules27238422

**Published:** 2022-12-01

**Authors:** Menghan Zhang, Yun Lu, Lu Sheng, Xinxin Han, Liming Yu, Weihua Zhang, Shangfeng Liu, Yuehua Liu

**Affiliations:** 1 Shanghai Key Laboratory of Craniomaxillofacial Development and Diseases, Shanghai Stomatological Hospital, Fudan University, Shanghai 200001, China; 18111370002@fudan.edu.cn (M.Z.); luyun_dentist@163.com (Y.L.); shenglu_kq@fudan.edu.cn (L.S.); xxhan@sibs.ac.cn (X.H.); ylmphoebe@126.com (L.Y.); 17211370002@fudan.edu.cn (W.Z.); 2 Department of Orthodontics, Shanghai Stomatological Hospital, Fudan University, Shanghai 200001, China; 3 Department of Oral and Maxillofacial Surgery, Shanghai Stomatological Hospital, Fudan University, Shanghai 200001, China

**Keywords:** obstructive sleep apnea, molecular pathogenesis, pharyngeal muscle fatigue, signaling pathways

## Abstract

Obstructive sleep apnea (OSA) is a common syndrome that features a complex etiology and set of mechanisms. Here we summarized the molecular pathogenesis of OSA, especially the prospective mechanism of upper? airway dilator fatigue and the current breakthroughs. Additionally, we also introduced the molecular mechanism of OSA in terms of related studies on the main signaling pathways and epigenetics alterations, such as microRNA, long non-coding RNA, and DNA methylation. We also reviewed small molecular compounds, which are potential targets for gene regulations in the future, that are involved in the regulation of OSA. This review will be beneficial to point the way for OSA research within the next decade.

## 1. Introduction

Obstructive sleep apnea (OSA) is a clinical condition characterized by sleep-related recurrent upper airway obstruction, hypopnea and apnea, resulting in chronic intermittent hypoxemia (CIH) and sleep disorders [1]. It estimated that 936 million adults aged 30–69 years (men and women) have mild to severe obstructive sleep apnoea and 425 million adults aged 30–69 years have moderate to severe obstructive sleep apnoea globally. The number of affected individuals was highest in China, followed by the USA, Brazil, and India [2]. It is a highly prevalent disorder which has rapidly evolved into a major global public health burden, independently linked with the development and control of numerous cardiovascular and metabolic conditions including hypertension, coronary artery disease, stroke, heart failure, type 2 diabetes or on-alcoholic fatty liver disease [3]. Polysomnography, the gold standard for the diagnosis of OSA, is utilized to monitor the frequency of obstructive respiratory events (apneas and hypopneas) during sleep. The severity of OSA is defined by apnea– hypopnea index(AHI), persons with an AHI of 5 to 15, 16 to 30, or more than 30 events per hour are considered to have mild, moderate, or severe obstructive sleep apnea, respectively [2]. There is a wide range of treatment options for OSA, including surgical interventions, lifestyle modifications, drug control, continuous positive airway pressure (CPAP), oral appliances (OAs) and hypoglossal nerve stimulation (HGNS) [4]. However, although there were some improvements in some aspects of OSA, no revolutionary changes have emerged in the progress of diagnosis and clinical treatment. Therefore, we reviewed current knowledge about pathogenesis, molecular mechanism of OSA, and exploration of some new breakthroughs, thus develop novel ideas for OSA.

## 2. The Pathogenesis of OSA and Pharyngeal Muscle Fatigue

**Figure 1:**
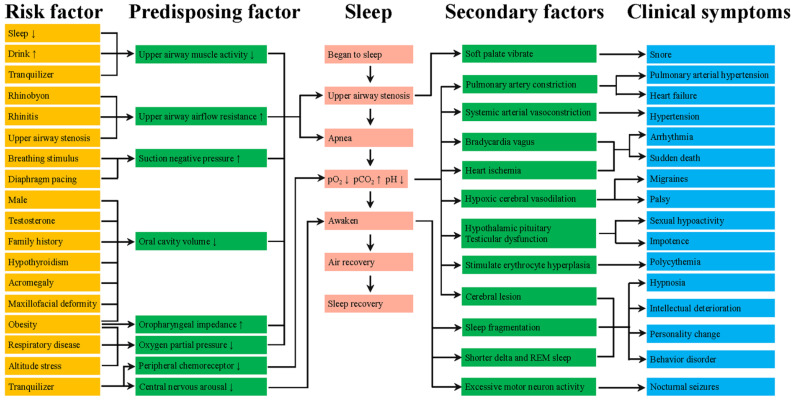
The pathogenesis of OSA.

The pathogenesis of OSA can be attributed to anatomical stenosis and pharyngeal dilator dysfunction (Figure 1). Anatomical stenosis includes upper airway anatomical structure stenosis, negative airway pressure and an increase in external tissue in pharyngeal space such as fatty tissue [5,6]. However, the pharyngeal collapse of OSA is partly due to stenosis of the upper airway anatomy [7]. The dysfunction of pharyngeal dilator might also play a key role in the pathophysiology of OSA [8,9]. Obesity can lead to soft tissue enlargement of the upper airway and craniofacial abnormalities, which are also important factors for the anatomical risk of OSA [10].

More details about the pathological process in OSA could be found in Figure 1. Among these, we are most interested in the pathogenesis of upper airway dilator neurological impairment. The basic mechanism of neuropathology in obstructive sleep apnea syndrome was controversial and single mechanism was unlikely to explain all the changes. The most reasonable explanation is that these changes reflect the effects of repeated exposure to hypoxia, vibration, abnormal movement, which may lead to local trauma caused by inflammation, impair nerve function by axonal injury, resulting in the vulnerability of motor nerve endings [11,12]. Therefore, these effects might induce upper airway muscle remodeling, alter contraction frequency and fatigue resistance of upper airway muscle [12,13]. Chronically, these changes make the airway narrower and easier to collapse. We think that active remodeling may help maintain muscle functions. 

## 3. OSA Correlated Signaling Pathway

**Figure 2:**
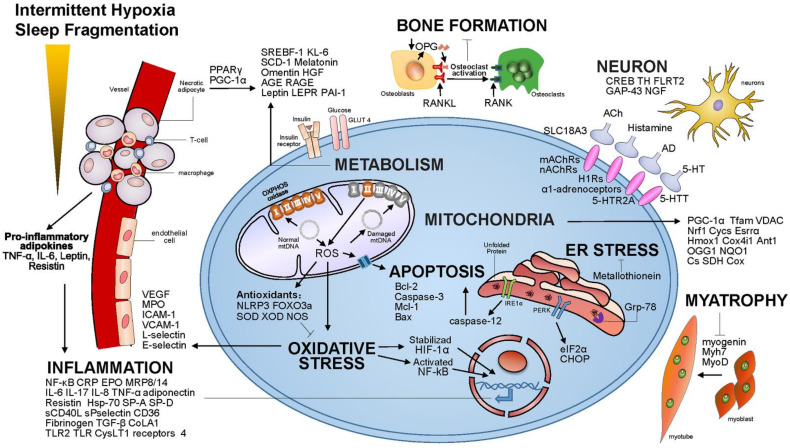
The signaling pathways involved in OSA.

With the development of modern molecular biology technology, research on the gene expression regulation of OSA has made rapid progress. Studies have shown that about 5 percent of human genes are associated with hypoxia, which works out to more than 1, 000 genes. It is known that OSA pathogenesis is related to a multifactorial process with a diversity of mechanisms, including oxidative stress, activation of the inflammatory response, endothelial dysfunction, metabolic alteration, and upper airway dilator neurological impairment [13,14]. We summarize the signaling pathways associated with the onset of OSA in Table 1 and list less than 100 OSA-related genes that were reported (Figure 2).

Intermittent hypoxia (IH) and sleep fragmentation (SF) are major pathophysiologic characters of OSA. IH acts as a trigger of oxidative stress, overt inflammation and increased cell apoptosis and neural activation, while SF is associated with a burst of neural activation and systemic inflammation [85]. IH activate a signaling cascade which leads to an unbalanced production of reactive oxygen species (ROS) and down-regulation of some endogenous antioxidants defense enzymes [77,86,87,88,89,90,91,92,93]. Prolonged oxidative stress disrupt important signaling pathways by activation of several transcription factors, contributing to inflammatory cascade, endothelial dysfunction and other adapts in OSA patients [94,95,96,97,98]. Otherwise, increases in systemic oxidative stress elicits the increase expression of pro-inflammatory cytokines and adhesion molecules associated inflammation responses pathway [98,99]. Moreover, mitochondrial dysfunction, one of the motivating factors for ROS, increased in OSA patients with abnormal structure accompanied by mitochondria DNA (mtDNA) damage, mitochondrial enzymes changes and respiratory metabolites disorder [100,101]. Long-term mitochondrial DNA damage as well as accumulation of mutations would lead to the dysfunction of Oxidative phosphorylation (OXPHOS) system, resulting in increased ROS production via complex I of the respiratory chain, which in return deteriorate mitochondria damage [93,100,102,103]. The endoplasmic reticulum (ER) stress is also involved in various OSA-associated pathologies [104,105]. Calcium homeostasis disturbances and/or unfolded proteins accumulation in the ER triggers the unfolded protein response (UPR). When ER stress intense, the UPR promotes cell death especially through activation of the pro-apoptotic transcription factor C/EBP homologous protein (CHOP), which was viewed as a major factors in triggering other damage related pathways [104]. Meanwhile, some genes related to apoptosis were upregulated in OSA patients since there was a positive correlation between the severity of sleep apnea and apoptotic cells [106]. Consistent evidences have also shown that chronic intermittent hypoxemia (CIH) have an effect on metabolic dysfunction such as lipid metabolism, insulin resistance and pancreatic beta cell dysfunction by regulatory enzymes of some metabolic and inflammatory parameters [98,106]. Studies have shown that plasma Alzheimer’s biomarkers are higher in patients with OSA than in the control group, and the mechanism of action may be related to sleep disturbances and nighttime hypoxia [107]. Some important pathways involved in OSA are also associated with higher prevalence of osteoporosis and neuromuscular dysfunction [10,108].

Although tremendous and complicated of OSA-associated signaling pathways have been reported, its genetic basis is still largely unknown. Now people are paying more attention to OSA-susceptibility genes and genetic polymorphisms [109,110,111]. One study [107] tried to identify novel biomarkers for OSA using systems biology approach. Genes pertaining to the top 10 pathways and used for Ingenuity Pathway Analysis. Twenty-three candidate genes were identified, out of which >30% of the genes were related to the genes involved in the neuron pathway (especially serotonin pathway) [107]. Nowadays, a few studies have focused on single nucleotide polymorphism (SNP) and loci. Wang et al. identified that local African ancestry at the chromosomal region 2q37 was significantly associated with AHI, and European and Amerindian ancestries at 18q21 suggestively associated with both AHI and saturation of blood oxygen (SaO2) < 90% [112]. Karla et al found a link between a single nucleotide polymorphism (SNP) in the region of apolipoprotein E (ApoE) and OSA status in children [113]; Gozal et al found a connection between a SNP in the p22 phox subunit of the NOX gene and cognitive deficits in children with OSA [114]; Researchers from 26 institutions have conducted genome-wide studies involved 12,558 participants in Hispanic/Latino Americans and identified two novel loci, which was associated with insulin signaling and Sterol-regulatory element binding proteins (SREBP) signaling, and refer to inflammatory, hypoxia signaling, and sleep pathways [115]. Future studies should focus on identifying the potential utility of the targeted genes.

## 4. MicroRNA (miRNA) in OSA

MicroRNA (miRNA) is a kind of non-coding RNA, which is widely used in organ development, inflammation, tumor development and other aspects because of its inhibitory effect on target genes. As OSA is a systemic disease, miRNA is bound to play an indispensable role in its occurrence and development (Table 2). Researchers indicate that the presence of endothelial dysfunction, atherosclerosis, and hypertension in OSA may be associated with up-regulations or down-regulations of some miRNAs [116,117,118,119]. Recent studies found that several miRNAs could influence IH process and affect hypoxia-induced cell apoptosis [120]. Some miRNAs up-regulated or down-regulated by hypoxia are direct targets of HIF-1α, HIF-2α, NF-κB, or their responsive genes, or some inflammatory signalings [121,122,123,124]. Therefore, it is suggested to identifying differentially expressed miRNAs and their potential spots in order to understand mechanism of OSA with targeted therapies. At present, although there have been some reports on the functional studies of miRNA in the OSA patients or animal models, systematic and in-depth studies on epigenetics still remain to be seen.

## 5. Long Noncoding RNAs (lncRNAs) in OSA

Long noncoding RNAs (lncRNAs), a novel class of non-coding RNAs, which function in regulating gene expression [136,137], affect numerous cellular processes [82] and are implicated in multiple diseases such as liver disease, cancer, and psychiatric disease [136,138,139]. Regarding lncRNAs in OSA, researchers are now at the initial and tentative launching stages. A well-established CIH rat model was used to conduct lncRNA microarray experiments on the heart samples of rats with CIH and under normoxia control. A total of 157 lncRNAs were upregulated and 132 lncRNAs were downregulated in a rat model of CIH compared with a sham control [140]. More details could be found in Table 3.

## 6. DNA Methylation in OSA

Very few studies have so far focused on the role of DNA methylation in OSA, which might bridge the gap in the molecular mechanisms underlying the pathophysiology of OSA. Studies to explore the potential association of DNA methylation patterns with the disease severity in the adult population with OSA are starting to emerge [144,145,146]. More details are found in Table 4. Further studies are required to elucidate the role of DNA methylation as a potential biomarker in the context of OSA.

## 7. Chemical Compounds for OSA Treatment

Because of their many unique natural advantages, small molecular compounds are of great significance in regulating OSA and mechanism research. Most of these small-molecule compounds are important gene inhibitors or activators of OSA-correlated signaling pathways (Table 5). These chemical compounds are mainly targeted with signaling pathways that include oxidative stress, apoptosis, mitochondria, inflammation, metabolism, and neuro-muscular connection [154,155,156,157,158,159,160,161,162,163,164,165,166]. Some clinical trials were aimed at evaluating the potential benefits of melatonin, which is a hormone that regulates sleep patterns; these benefits include being a potent antioxidant, reducing chemoreflex sensitivity, stabilizing ventilatory control, and reducing OSA severity. This clinical trial is registered with www.clinicaltrials.gov (accessed on 2 October 2022) (NCT02484300, NCT05309681). Other trials were aimed at exploring the benefits of Venlafaxine, which is an agent that increases the respiratory arousal threshold (neural drive) based on the hypothesis that OSA patients with a low arousal threshold may wake up too early before upper airway muscles can be activated to achieve stable ventilation. This clinical trial was registered with www.clinicaltrials.gov (accessed on 2 October 2022) (NCT02714400, NCT00084669). There are also some clinical trials targeted toward orexin and investigating the effects of ACT-541468, which is an orexin receptor antagonist against nighttime respiratory function in patients with mild-to-moderate obstructive sleep apnea. This clinical trial was registered with www.clinicaltrials.gov (accessed on 2 October 2022) (NCT03765294, NCT02841709).

## 8. Conclusions and Perspectives

1. The research on the signaling pathway and the popularization of rapid clinical diagnosis suggest that new small-molecule targeted drugs will be developed and applied rapidly in the next decade. Although the clinical diagnosis of OSA was recently standardized and the clinical treatment of OSA has been progressing rapidly, the relevant small-molecule targeted drugs have not made important progress due to our insufficient understanding of the signaling pathways involved in this disease, including the epigenetic pathways.

2. The field of epigenetics has attracted much attention in the past few years as a potential mechanism for the etiology and phenotypic variation of multiple diseases. Recent studies on the epigenetics of OSA phenotype expression further attest to the complexity of OSA and provide inspiring prospects for controlling OSA and its consequences with more individualized diagnosis and treatment methods. For example, if OSA is the cause of epigenetic changes in a gene, such a change might reverse after treatment of OSA, and may require incremental therapies that specifically target the epigenetic modification. Future research should focus on genome-wide association methods to identify epigenomic characteristics associated with certain phenotypes, which will help to provide new diagnostic biomarkers and targeted therapy for genetically susceptible individuals.

3. For the establishment of an OSA model, we need to simulate the pathogenesis of OSA in a manner that is as close to reality as possible. The electrophysiological states of the upper airway dilator muscle are diverse in waking and the different stages of sleeping and are also associated with sleep-related genes. As such, how can we get closer to the real OSA model? As far as we know, the OSA model of non-human primates has been seldom reported, except for earlier studies. We believe that the OSA model in non-human primates is of great significance to the study of the relevant pathogenesis, targeted drug screening, and therapeutic device development.

4. Although upper airway stenosis can be expanded by surgery, the relevant soft tissue research is still in the early stage. Targeted drug therapy and functional rehabilitation of the genioglossus muscle are likely to be an important direction regarding OSA in the future. We can expect to place these drugs in these oral appliances and treat OSA with a slow-release gel, which can additionally improve the function of an upper airway dilator.

5. We believe that among the related genes, it is more important to study those involved in nerve and muscle regulation. The study of these genes will make it easier to find a breakthrough in the treatment of OSA. For example, genes related to mitochondrial function include Hmox1, Cs, Cox4i1, Ant1, 8-OGG1, and NQO1.

6. Summary: OSA, as a representative of human systemic diseases whose hypoxia mechanism can be attributed to anatomical stenosis and pharyngeal dilator dysfunction, has the above characteristics of systemic diseases and is enough to trigger (or influence) various diseases. Therefore, we should pay more attention to the main molecular mechanisms of OSA pathogenesis when referring to the treatment, and thus, to effect a cure or prevent the occurrence of OSA. Preventive and therapeutic drugs targeting the relevant molecular targets are expected. We remain optimistic about the treatment of OSA in light of the current progress and OSA will be alleviated within decades.

## Figures and Tables

**Table 1:** Genes involved in OSA.

Signaling Pathway	Gene	Main Function in OSA
Oxidative stress	HIF-1α	HIF-1α was upregulated in patients with OSA. HIF-1α can enhance types I, Ⅱa, and Ⅱx fiber generation during the process of myogenic differentiation and suppress Ⅱb fiber generation [15].
	VEGF	VEGF was reported to be increased in the serum and plasma of OSA patients. Serum levels of VEGF are elevated in severely hypoxic patients with OSA and are related to the degree of nocturnal oxygen desaturation. This might constitute an adaptive mechanism to counterbalance the emergence of OSA-related cardiovascular disease [16].
	MPO, ICAM-1, VCAM-1, L-selectin, E-selectin	The increase in ICAM-1, VCAM-1, L-selectin, E-selectin, and MPO in peripheral blood is one of the mechanisms of cardiovascular damage in elderly patients with OSA [17,18].
	ROCK1, ROCK2	OSA patients showed significantly lower PBMC ROCK1 and ROCK2 levels than healthy controls in the morning but not in the evening [19].
	TNFα,EN-1	EN-1 and TNF-α gene expression levels were significantly higher in the OSA group than in the control group [20].
	NADPH oxidase	Long-term IH increased NADPH oxidase gene and protein responses in wake-active brain regions [21].
	NOS	Circulating nitric oxide is suppressed in OSA. Baseline endothelial expression of eNOS and phosphorylated eNOS were reduced in patients with OSA compared with control subjects [22,23,24,25].
	NLRP3, FOXO3a, SOD, XOD	miR-155 might be a positive regulator of the NLRP3 pathway by inhibiting the targeted FOXO3a gene. Chronic OSA also strikingly increased NLRP3, SOD, and XOD [26].
Apoptosis	Bcl-2, Caspase-3	Bcl-2 and cleaved caspase-3 play a critical role in underlying memory deficits in obstructive sleep apnea (OSA)-associated IH, and were upregulated after chronic IH treatment [27].
	Mcl-1, Bax	Hypoxia upregulated the anti-apoptotic Mcl-1 and downregulated the pro-apoptotic Bax. IH induced ERK1/2 and p38 MAPKs phosphorylation, whereas SH induced only p38 MAPK phosphorylation [28].
Mitochondria	MnSOD	The data from the in vitro and in vivo experiments indicate that CIH-mediated mitochondrial oxidative stress may play a major role in neuronal cell loss and neurocognitive dysfunction in OSA. Overexpression of MnSOD decreased CIH-mediated cortical neuronal apoptosis [29].
	PGC-1α, Tfam, VDAC	The expressions of PGC-1α, Tfam, and VDAC were higher in inner ear sensory hair cells in the CIH group, and there is an association between OSA and mitochondria [30].
	Nrf1, Cycs, Esrrα	Levels of mRNAs were implicated in mitochondrial biogenesis based on quantitative real-time RT-PCR performed on RNA isolated from genioglossus muscle from three groups [31].
	Hmox1, Cox4i1, Ant1, OGG1, NQO1, Cs	The mRNA levels of genes related to mitochondrial function, such as Hmox1, Cs, Cox4i1, Ant1, 8-OGG1, and NQO1, were all markedly lower in the genioglossus of the CIH group [31].
	SDH, Cox	Stained genioglossi sections demonstrated a lower number of Cox- and SDH-positive muscle fibers and reduced intensity of SDH and Cox staining in the CIH group [32].
ER stress	Grp-78, caspase-12, CHOP	Upregulation of Grp-78, caspase-12, and CHOP occurred after IH treatment, which was prevented by the injection of TUDCA [27].
	CHOP, eIF-2a	Protection of eIF-2 phosphorylation with systemically administered salubrinal throughout hypoxia/reoxygenation exposure prevented CHOP/GADD153 activation in susceptible motoneurons. The augmentation of eIF-2a phosphorylation minimizes motoneuronal injury in hypoxia [33].
	MT	M MT protection from ER-stress-induced apoptosis was mediated by upregulating Akt phosphorylation since the inhibition of Akt phosphorylation abolished MT’s protection from ER stress and apoptosis [34].
Inflammation	p50, p52, p65, c-REL, REL B, NF-kB	Chronic IH ability to induce cardiac ER stress, cell death, and inflammation can be prevented by MT, probably via upregulation of the Akt function [35,36].
	CRP	The increase in serum hs-crp content is closely related to the inflammation degree of OSA patients, which can promote the synthesis and release of chemokines and induce the expression of adhesion molecules in vascular endothelial cells to some extent, thus causing damage to the cardiovascular system. The SNP of CRP is correlated with hypertension in OSA patients [37].
	IL-6	Levels of IL6 were increased in the serum of OSA patients. The serum IL-6 level can be decreased in OSA patients using an effective treatment [38].
	IL-17	Vitamin D deficiency in patients with severe OSA is common with a negative association between IL-17 and vitamin D serum levels [39].
	IL-8	IL-8 precedes the development of systemic inflammatory markers in young children with sleep-related CIH [40].
	TNFα	TNFa was elevated in OSA patients [41].
	EPO	EPO is activated solely in response to hypoxia and, therefore, represents a better marker for HIF-1 activation [42].
	SP-A, SP-D	OSA pathogenesis was associated with changes in SP-A and SP-D decreased expression levels [43].
	TLR2, TLR4	OSA is associated with enhanced expression and signaling events downstream of TLR2 and TLR4 in circulating monocytes [44].
	Resistin	Resistin production can be enhanced by hypoxic stress during sleep, possibly mediating systemic inflammatory processes [45].
	CysLT1 receptors	CysLT1 receptors play a regulatory role in the pathogenesis of OSA in children [46].
	MRP8/14	Plasma MRP8/14 levels are associated with pediatric OSA and may reflect an increased risk for cardiovascular morbidity [47].
	sCD40L, sPselectin	Serum levels of sCD40L and sP-selectin are elevated in patients with moderate-to-severe OSA [48].
	CD36	In CIH-exposed mice that closely mimic the chronicity of human OSA, the increased accumulation and proliferation of pro-inflammatory metabolic M1-like macrophages highly expressing CD36 emerged in the aorta [49].
	Fibrinogen	Fibrinogen levels were significantly elevated in patients with severe OSA. Fibrinogen levels were directly related to AHI and the arousal index and inversely related to the mean and lowest oxygen saturation during sleep [50].
	Hsp-70	Hsp-70 was upregulated by repetitive hypoxemia in OSA and may be involved in the development of the atherogenic process in OSAHS [51].
	TGF-β, CoLA1	Serum TGF-β level was lower in OSA patients [52]. OSA can accelerate the progression of pulmonary remodeling through TGF-β/miR-185/CoLA1 signaling [53].
Metabolism	KL-6	Circulating KL-6 is a biomarker of lung injury in OSA [54,55].
	SREBF-1, SCD-1	CIH induces fasting dyslipidemia in both lean and obese mice due to the activation of SREBF-1 and SCD-1 [56,57,58]. In human subjects, hepatic SCD mRNA levels correlate with the degree of nocturnal hypoxemia [58].
	Melatonin	Circulating melatonin levels are elevated in OSA patients [59].
	Omentin	Circulating omentin levels are elevated in OSA patients and seem to be involved in the pathogenesis of OSAS [59,60].
	HGF	Combined detection of serum HGF concentrations in patients with OSA has a clinical value in judging the condition and curative effect and evaluating the cardiovascular damage [61].
	AGE, RAGE	AGEs may play an important role in insulin resistance in OSA and serve as a biomarker for patients with OSA with a high risk of type 2 diabetes mellitus [62,63,64,65].
	Leptin, LEPR	OSA patients have significantly higher levels of leptin. Leptin affects the sleep architecture, ventilation, and the defense of upper airway patency. The association between leptin and leptin receptor gene polymorphisms and susceptibility to OSA remains poorly defined due to conflicting data [66,67,68,69].
	PPARγ	PPARγ was downregulated in subjects with OSA [70].
	PAI-1	PAI-1 was significantly higher in subjects with OSA. Gene set enrichment analysis (GSEA) identified several gene sets that are upregulated in the adipose tissue of OSA patients, including the pro-inflammatory NF-κB pathway and the proteolytic ubiquitin/proteasome module [71].
Myatrophy	Myh7	Myh7 were both downregulated in palatopharyngeal tissues from OSA patients [72].
	MyoD, myogenin	The MyoD and myogenin mRNA in the CIH group was significantly lower compared with the control. When the oxygen level was normal, the myosin heavy chain (MHC), myogenin, and MyoD expression increased [73].
Bone formation	OPG/RANKL	The serum level of OPG and the OPG/RANKL ratio were lower in the OSA group [74].
	VDR	A low vitamin D serum concentration was reportedly linked to OSA susceptibility [75].
Neuron	5-HTR2A, 5-HTT	5-HT activity is required to maintain upper airway stability in OSA models. 5-HTR2A and 5-HTT genes may be susceptible markers to develop for OSA [76,77].
	H1Rs	Histamine excited HMN with an inward current under a voltage clamp and a depolarization membrane potential under a current clamp via H1Rs. This contributes an excitatory drive to the GG muscle involved in the pathogenesis of OSA [78].
	mAChRs	The mAChRs mechanism linked to GIRK channels would suppress HM activity, largely in REM sleep [79].
	nAChRs	The nAChRs activation on HMNs may contribute to the central maintenance of upper airway patency and prevent airway obstruction [80].
	α1-adrenoceptors	Chronic IH increases the noradrenergic drive to XII motoneurons including the sprouting of noradrenergic terminals in the XII nucleus and increased expression of α1-adrenoceptors [81].
	CREB	IH induced significant decreases in Ser-133-phosphorylated CREB without changes in the total CREB [82].
	GAP-43, TH, NGF	GAP-43, TH, and NGF were highly expressed in OSA groups. OSA can accelerate the progression of pulmonary remodeling through TGF-β/miR-185/CoLA1 signaling [83].
	SLC18A3, FLRT2	SLC18A3 gene expression was significantly upregulated in peripheral blood from patients with OSA, while FLRT2 was significantly depressed in patients with severe OSA [84].

**Table 2:** miRNAs in OSA.

Genes	miRNA	Function in OSA
Unknown	miR-664a-3p	miR-664a-3p levels are positively associated with AHI, LOS, and CIMT, and thus, it has a possible role in the pathogenesis of atherosclerosis in OSA patients and as a noninvasive marker of these related conditions [125].
GAX	miR-130a	miR-130a may be involved in the progression of OSA-associated PHT by downregulating the GAX gene [126].
Unknown	miR-223	CIH decreased the expression of miR-223, whereas 2-methoxyestradiol reversed the downregulation of miR-223, both in vivo and in vitro [127].
CoLA1	miR-185	OSA could activate the expression of TGF-β, which subsequently suppressed miR-185 and promoted CoLA1 expression [83,128].
Smad3	miR-145	miR-145/Smad3 signaling pathway might promote aortic remodeling during OSA [128].
Nrf2, AMP kinase, and tight junction pathways	miR-630	The expression of exosomal miRNA-630 was reduced in children with endothelial dysfunction and was normalized after therapy, along with restoration of endothelial function [129].
Autophagy and apoptosis	miR-16, miR-718, miR-1249, miR-193, miR-218, miR-30B	Four (miR-1249, miR-193, miR-218, and miR-30B) were upregulated and two (miR-16 and miR-718) were downregulated markedly in CIH [130].
Beclin-1	miR-30a	Suppression of miR-30a via the expression of the antisense of miR-30a significantly increased Beclin-1 levels to enhance endothelial cell autophagy in vitro and in vivo, which improved endothelial cell survival against CIH [131].
Unknown	miR-26b, miR-207	miR-26b and miR-207 could be involved in OSA-induced cognitive impairments [122].
PANK CAD	miR-107, miR-485-5p, miR-574-5p, miR-199-3p	These different microRNAs also play a significant role in metabolism, hypoxia, and oxidative stress, and might participate in OSA [119].
Bcl-2	miR-34a-5p	The overexpression of miR-34a-5p activated Beclin 1 through Bcl-2 inhibition in CIH and participated in CIH-induced autophagy [132].
XIAP	miR-146a-5p	miR-146a-5p could attenuate viability and promote the apoptosis of H9c2 by targeting XIAP, thus aggravating the H9c2 cell injury induced by IH [133].
Unknown	miR-126-3p, let-7d-5p, miR-7641, miR-1233-5p, miR-320b, miR-145-5p, miR-107, miR-26a-5p	miR-145-5p and let-7d-5p in combination can identify healthy OSA, and the presence of miR-126-3p, miR-26a-5p, and miR-107 was strongly indicative of OSA with arterial hypertension [134].
FOXO3a	miR-155	miR-155 might be a positive regulator of the NLRP3 pathway by inhibiting the targeted FOXO3a gene [135].

**Table 3:** The lncRNAs involved in OSA.

lncRNAs	Function in OSA
lncRNA-CPS1-IT	CPS1-IT was downregulated in an OSA rat model. Overexpressed CPS1-IT increased the activity of NO, NOS, and SOD, as well as α-SMA expression, whereas decreases in LPO activity, PCNA expression and IL-1β expression occurred through NF-κB signaling pathway via inhibiting the HIF1 transcriptional activity [141].
lncRNA-ROR	lncRNA-ROR revealed properties that are useful for regulating the hypoxia response. CoCl_2_ increased the expression of ROR. ROR overexpression upregulated the anti-apoptotic protein Bcl-2; decreased p53, Bax, cleaved caspase-3, miR-145, and the phosphorylation of MAPK; and increased the expression of HIF-α and the phosphorylation of ERK [142].
XR_596701, XR_344474,XR_600374, ENSRNOT00000065561, XR_590196, XR_597099	Three lncRNAs (XR_596701, XR_344474, and ENSRNOT00000065561) increased and three lncRNAs (XR_600374, XR_590196, and XR_597099) decreased in the heart samples of rats exposed to eight weeks of CIH [143].

**Table 4:** DNA methylation in OSA.

Target Genes	Function in OSA
AR, NPR2, L1R2, SP140	OSA-related hypoxia leads to the altering in the promoter methylation of AR, NPR2, L1R2 and SP140 [147,148].
FOXP3	The FOXP3 gene, which regulates expression of T regulatory lymphocytes, ismore likely todisplay increasedmethylation among children with OSA who exhibit increased systemic inflammatory responses [149].
eNOS	A CpG site showed significantly higher methylation levels. eNOS mRNA expression levels were significantly reduced [150].
AOEs	Long term IH (IH) increased DNA methylation of genes encoding AOEs. Treatment with decitabine, a DNA methylation inhibitor, prevented DNA methylation, normalized the expression of AOE genes and ROS levels [151].
Rab3a	Mice engrafted with TC1 epithelial lung cancer cells and controls were exposed to IH. Increased Rab3a showed significant plasma cirDNA modification, increasing tumor invasion [152].
Ace1, Atg	IH-exposed mice showed higher lever of DNA methylation patterns of the Ace1 and the Agt genes CD31+ endothelial cells [153].

**Table 5:** The chemical compounds involved in OSA.

Targets	Chemical Compounds	Main Functions in OSA
Nox1 and Nox4	GKT137831	Nox1 and Nox4 inhibitor [153].
ROS scavenger, antioxidant, anti-inflammatory, and mucolytic effects	NAC	Limiting ROS production by NAC could suppress ER stress activation [155].
RhoA inhibitor	Y27632	Treatment with Y27632 reduced both Systolic blood pressure and renal sympathetic nerve activity in rats exposed to chronic IH [156].
Lipid-lowering medicine	Statin	Inhibition of the inflammatory response by statins may be due to the down-regulation of TLR4 and TLR2 expression, there by reducing the release of downstream effectors [167].
TLR2 and TLR4	Candesartan	TLR2 and TLR4 expression at mRNA and protein levels are inhibited by candesartan both in vitro and in vivo [168].
CysLT1 receptors	LTD4	LTD4 can promote T cell proliferation in adenoid tissues via activation of CysLT1 receptors in children with OSA [47].
Antioxidant and anti-inflammatory	ALA	ALA attenuates endothelial dysfunction by preventing oxidative stress and inflammation and restoring nitric oxide bioavailability in mice exposed to CIH [169].
NOS inhibitor	ADMA	Nasal CPAP improves endothelial function, in part by the decreasing ADMA concentration, thereby potentiating NO production [170].
Inhibits cyclic guanosine monophosphate-specific phosphodiesterase 5.	Sildenafil	In patients with severe obstructive sleep apnea, a single 50-mg dose of sildenafil at bedtime worsens respiratory and desaturation events [171].
Norepinephrine reuptake inhibitor antimuscarinic	Atomoxetine, oxybutynin	A combination of noradrenergic and antimuscarinic agents administered orally before bedtime on one night greatly reduced OSA severity [172].
An inhibitor of NET and SERT, and prevents the reduction in genioglossus activity	Desipramine	Desipramine reduces the state-related drop in tonic genioglossus muscleactivity that occurs from wakefulness to non-REM sleep and reduces airway collapsibility [173].
AD, as an adipocyte-specific protein, regulates metabolism	AD	Impaired mitochondrial structure and function was significantly improved and a percentage of type I fiber was elevated. Moreover, a significant decrease in phosphorylation of LKB1, AMPK, and PGC1-α, whereas there was significant rescue of such reduction in phosphorylation [174].
TUDCA and 4-PBA, which are two chemical chaperones that reduce ER stress by facilitating proper protein folding	TUDCA, 4-PBA	Attenuators of ER stress may serve as novel adjunct therapeutic agents for ameliorating OSA-induced neurocognitive impairment [175].
A specific inhibitor of MEK1/2 and blocks ERK1/2 activation of a competitive p38MAPK inhibitor	U0126, SB202190	Both ERK and p38MAPK inhibitors attenuated the IH-induced Mcl-1 increase. In SH, only p38MAPK inhibition decreased Mcl-1 expression [176].
E_2_ and RD inhibited the overexpression of HIF-1α	E_2_, RD	ERα may be responsible for downregulation of HIF-1α by E_2_ or RD via activation of downstream p38 MAPK pathways [167].
miR-223	2-methoxyestradio	CIH decreased the expression of miR-223, whereas 2-methoxyestradiol reversed the downregulation of miR-223 both in vivo and in vitro [127].
AChEI	Donezepil, Physostigmine	A cholinesterase inhibitor, promotes cholinergic transmission [177,178].
Nicotinic agonist	1,1-dimethyl-4-phenylpiperazinium iodide	Excited hypoglossal motoneurons via a Ca^2+^-sensitive and TTX-insensitive inward current [80].
The alpha1 receptor antagonist	terazosin	Provides noradrenergic activation and significantly decreases GG activity in wakefulness and non-REM sleep [179].
Adenylyl cyclase activator	Forskolin	Increases cAMP at the HMN, as well as respiratory-related and tonic genioglossus activities, during wakefulness and non-REM sleep but not REM sleep [180].
A weak SSRI	Trazodone	Simultaneously inhibits SERT, 5-HT2A, and 5-HT2C receptors, reduces levels of serotonin thus improve apnea and hypopnea episodes in OSA patients [163].
SSRI	Paroxetine	Block 5-HT re-uptake, can increase the peak sleep inspiratory velocity and the activity of genioglossal muscle in OSA patients [181].
Serotonin antagonists	Methicillin, Ritanserin	Reductions in plasma 5-HT levels, and induced apnea [164,165].
Non-selective CB1/CB2 receptor agonist	Dronabinol	Reduced the frequency of spontaneous central apneas in a rodent model of sleep-related breathing disorder [166].
The carbonic anhydrase inhibitor	Acetazolamide	Acetazolamide improves sleep apnea at high altitude by decreasing AHI and percentage of periodic breathing time and increasing nocturnal oxygenation [15].

## Data Availability

Not applicable.
